# Inactivated *E. coli* transformed with plasmids that produce dsRNA against infectious salmon anemia virus hemagglutinin show antiviral activity when added to infected ASK cells

**DOI:** 10.3389/fmicb.2015.00300

**Published:** 2015-04-16

**Authors:** Katherine García, Sebastián Ramírez-Araya, Álvaro Díaz, Sebastián Reyes-Cerpa, Romilio T. Espejo, Gastón Higuera, Jaime Romero

**Affiliations:** ^1^Laboratorio de Biotecnología, Unidad de Alimentos, Instituto de Nutrición y Tecnología de los Alimentos, Universidad de ChileSantiago, Chile; ^2^Facultad de Química y Biología, Centro de Biotecnología Acuícola, Universidad de Santiago de ChileSantiago, Chile; ^3^Centro Nacional de Genómica y Bioinformática (Omics Solutions)Santiago, Chile

**Keywords:** double-stranded RNA, infectious salmon anemia virus, bacterial delivery, antiviral, aquaculture

## Abstract

Infectious salmon anemia virus (ISAV) has caused great losses to the Chilean salmon industry, and the success of prevention and treatment strategies is uncertain. The use of RNA interference (RNAi) is a promising approach because during the replication cycle, the ISAV genome must be transcribed to mRNA in the cytoplasm. We explored the capacity of *E. coli* transformed with plasmids that produce double-stranded RNA (dsRNA) to induce antiviral activity when added to infected ASK cells. We transformed the non-pathogenic *Escherichia coli* HT115 (DE3) with plasmids that expressed highly conserved regions of the ISAV genes encoding the nucleoprotein (NP), fusion (F), hemagglutinin (HE), and matrix (M) proteins as dsRNA, which is the precursor of the RNAi mechanism. The inactivated transformed bacteria carrying dsRNA were tested for their capacity to silence the target ISAV genes, and the dsRNA that were able to inhibit gene expression were subsequently tested for their ability to attenuate the cytopathic effect (CPE) and reduce the viral load. Of the four target genes tested, inactivated *E. coli* transformed with plasmids producing dsRNA targeting HE showed antiviral activity when added to infected ASK cells.

## Introduction

In recent years, the Chilean salmon farming industry has grown into one of the major salmon producers worldwide. However, the appearance of the infectious salmon anemia virus (ISAV) has strongly affected the salmon industry, resulting in high mortality and substantial economic losses (García et al., 2013). ISAV belongs to the genera Isavirus from the *Orthomyxoviridae* family (Krossøy et al., 1999; Palese and Shaw, 2007). It possesses a segmented genome of single-stranded RNA with negative polarity (Kulshreshtha et al., 2010) that encodes eight structural proteins and two non-structural proteins (Cottet et al., 2011). Infection with ISAV principally affects Atlantic salmon and other salmonid species (Raynard et al., 2001), causing systemic disease. The clinical and pathological signs of the disease can result in death (Evensen et al., 1991), causing a strong decline in production with serious economic costs. These losses are due to the limited availability of effective vaccines and specific antiviral treatments (García et al., 2013). To date, no effective pharmacological treatment has been developed for this disease, and the success of prevention strategies against ISAV is uncertain.

Considering the limitations of existing treatments, a plausible solution is the use of RNA interference (RNAi), which is a very promising alternative against viral diseases (Peng et al., 2005; Sarathi et al., 2008b; DeVincenzo et al., 2010). RNAi is an intracellular process by which small interfering RNAs (siRNAs) direct the degradation of matching messenger RNA (mRNA) (Hammond et al., 2000; Provost et al., 2002; Hannon and Conklin, 2004; Tijsterman and Plasterk, 2004; Gregory et al., 2005), preventing its translation into protein and causing gene silencing (Hammond, 2005). Effective delivery to the appropriate cells remains a major obstacle to successful RNAi. Several strategies exist, from the delivery of naked siRNA duplexes to more complex methods, including the systemic delivery of siRNA complexes as conjugates (La Fauce and Owens, 2012) and the use of non-pathogenic bacteria for delivery of dsRNA (Sarathi et al., 2008a). Each approach has its advantages and disadvantages, which must be considered when choosing a delivery strategy. Recently, the development of RNAi therapies for viral disease treatment has been suggested for aquatic organisms (Lima et al., 2013). Successful reports have been published on studies using RNAi technology against white spot syndrome virus (WSSV) (Sarathi et al., 2008a,b), CyHV-3 virus (Gotesman et al., 2014) and the virus that causes hemorrhagic septicemia, VHSV (Ruiz et al., 2009; Kim and Kim, 2011; Kim et al., 2012). VHSV, like ISAV, has a single-stranded RNA genome of negative polarity (Kulshreshtha et al., 2010), which makes this strategy very encouraging for our purposes.

In this work, we hypothesized that blocking the expression of four structural viral genes, nucleoprotein (NP), which is bound in multiple subunits to the genomic RNA (Palese and Shaw, 2007), matrix protein (M), which constitutes the most abundant structural protein (Falk et al., 2004; Palese and Shaw, 2007), hemagglutinin (HE) (Falk et al., 2004; Müller et al., 2010) and fusion (F) (Falk et al., 2004; Palese and Shaw, 2007), which are the surface glycoprotein, would circumvent ISAV replication. To this end, we explored the use of bacterially synthetized dsRNAs directed against NP, F, HE, and M to prevent the synthesis of these proteins by specific degradation of their respective mRNAs and decrease the viral load and attenuation of CPE in ASK cells.

## Materials and methods

### Cell culture and virus

Atlantic salmon kidney cells (ASK) were acquired from the ATCC (ATCC® CRL-2747) (Devold et al., 2000). Monolayers of ASK were maintained at 15°C in Leibovitz medium (L-15, Gibco) supplemented with 10% fetal bovine serum (FBS, Gibco), penicillin (100 U/ml, Gibco), streptomycin (100 μg/ml, Gibco), gentamycin (50 μg/ml, US Biological) and β-mercaptoethanol (55 mM, Bio-Rad, USA). A viral inoculum stock of ISAV genotype HPR35, with a titer of TCID50 = 10^7^/mL, was kindly provided by ADL diagnostics. The virus was propagated in monolayers of 70% confluent ASK cells as described in the viral infection section. All supernatants of the culture (aliquots of virus) were stored at −80°C.

### Viral infection

ASK cells were grown in 6-well plates to 70% confluence. At this point, the cells were infected with a 1/10 dilution of viral stock (TCID_50_ = 10^7^) prepared in non-supplemented L-15 medium. The virus was allowed to adsorb for 4 h. Then, the monolayer was washed twice with phosphate-buffered saline (PBS), and supplemented L-15 was added. The cells were incubated at 15°C for 11 days post-infection (dpi) until the cytopathic effect (CPE) was observed (Dannevig et al., 1995). At the end of infection, supernatants of the culture were harvested and stored at −20°C for further analysis. The CPE observed during infection was followed using an inverted Motic model EA31 microscope. Five visual fields per well were recorded using a Motic cam 2500 and Motic Image Plus 2.0 software.

### Identification of conserved sequences in ISAV genes

Reference genomic sequences of the ISAV NP (NC_006502.1), F (NC_006500.1), HE NC_006499.1), and M (NC_006497.1) genes were identified in NCBI databases (http://www.ncbi.nlm.nih.gov/genome). Each sequence was compared with the sequences in GenBank using *blastn* (Altschul et al., 1990); the *e-value* parameter was adjusted to 1.10e^−5^, and the search was restricted to sequences of the ISA virus taxa (txid: 55987) and Chilean isolates (Table 1). The remaining parameters used were the default values. Groups of identified sequences were aligned using ClustalX (Larkin et al., 2007), decreasing the penalties for the occurrence of spaces and using the default input parameters. All alignments were adjusted manually. Incomplete sequences or conflicts in sequencing were eliminated from the analysis. From the alignment, a consensus sequence of 500 bp was searched for each viral genomic sequence and selected for dsRNA design. Each of these sequences was compared with the *Salmo salar* database (Di Génova et al., 2011) to evaluate the *off-*t*arget* effects of the RNAi.

**Table 1:** **GeneBank accession of Chilean ISAV isolates sequences genes used for the alignments in this study**.

**Viral segment**	**GenBank accession**
Nucleoprotein	DQ520594.1; GU830905.1; GU830897.1
Fusion protein	FJ592146.1; FJ592163.1; EU449765.1; GU830899.1; FJ592162.1; FJ592165.1; EU130923.1; EU849005.1; EU849011.1; FJ592140.1; FJ592143.1; EU449768.1; EU849007.1; EU449767.1; EU552491.1; FJ592142.1; FJ592150.1; EU849006.1
Hemagglutinin protein	EU849018.1; GU830900.1; FJ594303.1; FJ594301.1; FJ594326.1; FJ594317.1; EU851043.1; EU271682.1; EU849012.1; EU849015.1; EU625681.1; EU625675.1; FJ594325.1; EU849017.1; EU625677.1; FJ594319.1; EU625668.1; FJ594296.1; EU625678.1; EU625674.1; FJ594323.1; EU625671.1; EU849016.1; JQ712975.1; EU849013.1; EU849014.1; FJ594297.1; EU625676.1; EU625669.1; EU625670.1; FJ594285.1; FJ594328.1; EU625667.1; FJ594292.1; FJ594332.1; EU625679.1; FJ594306.1; FJ594315.1; GU830908.1; EU65672.1; FJ594316.1; FJ594295.1; EU625666.1; FJ594294.1; EU625680.1; FJ594284.1; FJ477897.1
Matrix protein	AF312315.1; GU830902.1; GU830910.1

### Construction of dsRNA-producing vectors targeting NP, F, HE, and M

Total viral RNA was extracted from 200 μL of supernatants of ISAV-infected ASK cells using the Total RNA Kit I (Omega Biotek) according to the manufacturer's instructions. cDNA was synthetized as follows: 4 μL RNA plus 1 μL of random primer (0.5 mg/mL; Promega, USA) were heated for 5 min at 70°C. Immediately after this step, 15 μL of reverse transcription mix was added (dNTPs, MgCl_2_, RNasin, 5X buffer, Improm-II RT; Promega, USA), and the samples were incubated at 25°C for 5 min, 42°C for 60 min and 70°C for 15 min. To amplify the 500 bp conserved region that was previously selected, 3NP, 5F, 6HE, and 8M primers (Table 2), containing recognition sites for the *Sac*II or *Xho*I restriction enzymes, were used independently for PCR amplification. The following thermal profile was used: 1 cycle of 5 min at 95°C, 30 cycles of 30 s at 95°C, 30 s at 55°C, and 30 s at 72°C and a final extension of 10 s at 72°C. Subsequently, each PCR product was cloned into pGEMT easy (Promega, USA), cut simultaneously with the *SacII* (New England Biolabs, USA) and *XhoI* (New England Biolabs, USA) restriction enzymes and ligated into the L4440 vector (graciously donated by Cold Spring Harbor Laboratory, NY, USA), which contains T7 promoter sites flanking each side of the multiple cloning site (MCS). L4440 plasmids carrying NP, F, HE, or M fragments were sequenced in Macrogen (USA) to confirm the sequence and subsequently electroporated into *Escherichia coli* bacterial strain HT115 (DE3), an RNase III-deficient non-pathogenic strain (graciously donated by Cold Spring Harbor Laboratory, NY, USA).

**Table 2:** **List of the primers used in this study**.

**Primer**	**Experimental protocol**	**Primer orientation**	**Sequence**	**Product length (bp)**	**References**
3 NP	Cloning	Sense	CCGCGGAGCTTTCTGATTGACCCACCT	480	This work
		Antisense	CTCGAGAAGCATCTCCCTGATAGCGC		Cottet et al., 2010
5 F	Cloning	Sense	CCGCGGCCAAATGCGGGAGGAAAGGA	450	This work
		Antisense	CTCGAGCTCCTGGGAATGCTCTCTGG		This work
6 HE	Cloning	Sense	CCGCGGCTGCAGGCCAAAAACGGAAA	492	This work
		Antisense	CTCGAGGGTTCCCCTCACTTCAAAGGT		This work
8 M	Cloning	Sense	CCGCGGTGCTACTTACACTTGGCGGG	451	This work
		Antisense	CTCGAGCCCAGGAGCACCATCTTCTC		This work
3 NPi	PCR	Sense	CAATGGTTGCAACAGCATTC	205	This work
		Antisense	ACTTGCCAGCTTCGATCTGT		This work
5 Fi	PCR	Sense	ATCTGCGGAGGTACAACAGG	224	This work
		Antisense	ACCAGTACAGGCGATGGAAC		This work
6 HEi	PCR	Sense	ACTTGGGAACCAATGACTGC	182	This work
		Antisense	ACCGGTAATTGCGTCTGTTC		This work
8 Mi	PCR	Sense	GAAAGATCCACCGTCTGGAA	200	This work
		Antisense	TCTGCATCCTGCTGTGTAGC		This work
3 NPq	qPCR	Sense	ATGGCCGATAAAGGTATG	348	This work
		Antisense	TTGTTGTCAACCATGCCAC		This work
5 Fq	qPCR	Sense	CTGTTGCACTCAGCATGGAT	257	This work
		Antisense	CTCCTGGGAATGCTCTCTGG		This work
6 HEq	qPCR	Sense	GGCACGATTCATAATTTTATTCC	223	This work
		Antisense	TGAAGCAGATGAGTGGAAGG		This work
8 Mq	qPCR	Sense	TGGATACAAAAACATCTACCATGC	200	This work
		Antisense	TGGTTCAAGGTTTTGACTTTCAC		This work
Mx	qPCR	Sense	CTGGAGGAACCAGCAGTCAA	273	Abid et al., 2013
		Antisense	TAAGGGTCGGTCGTCTTCCT		
ILA	qPCR	Sense	GGCTATCTACCATGAACGAATC	155	Mjaaland et al., 2002
		Antisense	GCCAAGTGTAAGTAGCACTCC		
β-actina	qPCR	Sense	CCAAAGCCAACAGGGAGAAG	91	Olsvik et al., 2005
		Antisense	AGGGACAACACTGCCTGGAT		

### Production and quantitation of antiviral dsRNA

*E. coli* HT115 (DE3) carrying L4440 vectors with NP, F, HE or M inserts were grown in 10 mL of LB medium supplemented with 100 μg/mL ampicillin (Winkler, Chile). Induction of double-stranded RNA (dsRNA) was performed with 2 mM IPTG (Invitrogen, USA) for 4 h, and a total nucleic acid extraction was performed. The bacterial pellet was resuspended in 5 mL of 70% ethanol in PBS, incubated at room temperature for 5 min and collected by centrifugation at 6000 g for 5 min at 4°C. Then, the bacterial pellet was resuspended in 1 mL of 150 mM NaCl (Winkler, Chile) and incubated at room temperature for 1 h; the centrifugation was then repeated for 10 min. The supernatant was collected, and the genetic material was allowed to precipitate at −20°C overnight. Afterwards, the supernatant was centrifuged for 30 min at 13000 g at 4°C, and the formation of a white pellet was observed (Posiri et al., 2013). These nucleic acids were treated with 1 μL RQ1-RNase free DNase (Promega, USA) and 8 M lithium chloride (Winkler, Chile) to precipitate and remove any single-stranded RNA. Finally, the samples were treated with 8 M lithium chloride to precipitate the dsRNA. To finish, dsRNA was resuspended in 170 μL nuclease-free water (Winkler, Chile). The purified dsRNA was quantified using the DNA 1000 LabChip® kit on a Bioanalyzer Agilent 2100 (Agilent Technologies, USA). To verify the identity of cloned dsRNA, purification of each dsRNA from a 1% w/v agarose gel was performed. Each dsRNA (1 μL) was used to synthesize cDNA as described above. Subsequently, conventional PCR was performed using primers NPi, Fi, HEi, and Mi to amplify an internal section of the conserved regions of ISAV. The thermal profile used was 1 cycle of 5 min at 95°C, 30 cycles of 30 s at 95°C, 30 s at 55°C, and 30 s at 72°C and a final extension of 10 s at 72°C.

### Bacterial co-localization assay

ASK cells were seeded into a 24 well-plate at a density of 1 × 10^5^ cells/well. After 2 days, the cells were incubated for 40 min with the fluorescent probe CellTracker™ Orange CMRA (Invitrogen, USA) at a final concentration of 10 μM. Then, the cells were washed three times with PBS and treated with attenuated *E. coli* HT115 (carrying the green pBADT plasmid) overexpressing green fluorescent protein (GFP) in supplemented L-15 media at a multiplicity of infection (MOI) of 500 bacteria/cell (the maximum MOI that did not cause cell damage). Attenuation of the bacterial culture was performed by adding formaldehyde at a final concentration of 0.5% v/v and incubating for 20 min at room temperature. Immediately after this step, the bacterial culture was centrifuged and washed twice with PBS, quantified in a Petroff-Hausser chamber and used for analysis. The incubation of ASK cells with attenuated bacteria in supplemented L-15 media was followed by 4 days at 15°C. During the assay, slides with cell samples were removed at 0, 24, 48, and 96 h. Each slide was fixed with 4% paraformaldehyde (PFA) for 10 min and then treated with 50 mM NH_4_Cl for 10 min. Finally, the slides were mounted in 1,4-diazabicyclo[2.2.2]octane (DABCO) and visualized using a Nikon C2^+^ confocal microscope. Phase contrast photographs were taken using a Carl Zeiss LSM 510 confocal microscope with a DIC 3.4 filter.

### Antiviral treatment in ISAV-infected ASK cells

ASK cells were grown in 6-well plates to 70% confluence. Then, the cells were infected with ISAV HPR35 and treated individually with attenuated *E. coli* HT115 carrying dsRNA against HE, NP, M, or F individually or in a mixture of equal proportions. Additionally, a treatment with attenuated *E. coli* carrying an empty pL4440 vector was included. In all cases, we used a MOI of 500 (bacteria/cell) because it was the maximum MOI that did not cause cell damage. The attenuation process was the same as described above (in the bacteria co-localization assay). Cells that underwent different treatments were harvested at 11 dpi and incubated in EDTA-free protease inhibitor buffer (Roche, USA). These samples were used for real-time PCR and western blot analyses. In parallel, the supernatants were collected and used to determine the viral titer using a viral plaque assay. The CPE observed during each treatment was followed in an inverted Motic model EA31 microscope and recorded photographically using a Motic cam 2500; five visual fields were randomly analyzed with Motic Image Plus 2.0 software.

### Real-time qPCR analysis

The procedure for total RNA extraction and cDNA synthesis was the same as described above except we used primer pairs NPq, Fq, HEq, and Mq (Table 2) instead of random primers. To estimate the mRNA expression levels of NP, F, HE, and M, real time RT-PCR was carried out using the Corbett Rotor-Gene 6000 Thermal Cycler (Corbett Life Science) according to the manufacturer's instructions and using Rotor-Gene Version 1.7.87 software. All reactions were performed in triplicate. The PCR primer pairs NPq, Fq, HEq, Mq and the control gene β-actin (Olsvik et al., 2005) are shown in Table 2. The PCR reactions were performed in a final volume of 10 μL using Light Cycler® 480 SYBR Green I Master Mix (Roche, USA) with 1 μL of cDNA and 1 μL of each 50 mM primer. The thermal profile of real-time qPCR was as follows: hold for 1 cycle of 5 min at 95°C followed by 40 cycles of 15 s at 95°C, 10 s at 55°C and 10 s at 72°C and a final hold of 1 min at 40°C. Relative amounts of each viral transcript are expressed as the fold change in log_2_ of NP, F, HE, or M in infected cells treated with bacteria carrying antivirals compared to infected cells without antiviral treatment. To estimate the mRNA expression levels of Mx reactions were carried out under the same conditions previously mentioned. All reactions were performed in triplicate. All data were normalized to β-actin expression.

### Western blot analysis

Sodium dodecyl sulfate-polyacrylamide gel electrophoresis (SDS-PAGE) was performed using 15 μg of protein from each sample on a 4–12% NuPAGE Bis-Tris Gel (Bio-Rad, USA). The proteins were blotted onto polyvinylidene difluoride (PVDF) membranes, blocked with 5% dry milk and incubated at 4°C overnight with anti-HE antibody (clone 8D2/E9; GrupoBios, Bios-Chile) diluted 1:2000 or anti-β-actin antibody (clone AC-74, Sigma-Aldrich) diluted 1:500, followed by a 1 h incubation with the secondary antibody [rabbit-anti-mouse horseradish peroxidase (HRP), Invitrogen, USA] at a dilution of 1:20000. The blots were developed using Novex® ECL HRP Chemiluminescent Substrate (Invitrogen, USA) and exposed to Carestream Kodak Biomax Light film (sigma Aldrich, USA). The films were scanned and used for expression analysis with ImageJ 1.43 μ software.

### Viral plaque assay

The viral titre of the supernatant from ASK culture infected with ISAV HPR35; ASK infected and treated with attenuated *E. coli* HT115 carrying plasmid L4440 or ASK infected and treated with attenuated *E. coli* HT115 carrying dsRNA against HE, was determined with a plaque assay (Castillo-Cerda et al., 2014). ASK cells were seeded into 12-well plates and incubated for 3 days at 15°C. Then, the culture medium was removed from the monolayer, and 500 μL of supernatant containing virus, ten-fold serially diluted, from different conditions (ISAV HPR35, ISAV HPR35 infection + *E. coli* HT115 pL4440 or ISAV HPR35 infection + *E. coli* HT115 dsRNA HE) or 500 uL of supernatant from uninfected ASK culture (ASK control) were added. After 4 h at 15°C, the inoculum was removed, and 3 mL of semi-solid medium was added to each well. The plates were incubated for 10 days post-infection at 15°C. At day 10 post-infection, the cells were fixed in 1 mL of formalin (37%) for 1 h at 25°C, and the semi-solid medium was removed. For visualization, 2 mL of crystal violet (1%) was added for 1 h at 25°C, and the excess crystal violet was removed. Finally, the number of lysis plaques formed in wells was counted for each antiviral treatment.

### Statistical analysis

All experiments were performed in triplicate. The data were analyzed using GraphPad v. 5.01. Real-time PCR data were analyzed using Rest 2009 software. Protein expression data were analyzed using an ANOVA with Bonferroni's multiple comparison post-test. Viral plaque assay data were analyzed using the Wilcoxon test.

## Results

### dsRNA design and production

#### Identification of conserved regions of four genes involved in the viral cycle of ISAV (NP, F, HE, and M)

To appropriately design the dsRNA sequences, one 500 bp conserved region (Supplementary Table 1) was identified in the alignment of the Chilean ISAV isolates considered in this study (Table 1) for each of the four viral genes NP, F, HE, and M. The characteristics of these conserved sequences are described in Table 3. Note that the sequence identity (^*^) was the percentage of nucleotide of sequences that had the same residue at the same position in the alignment.

**Table 3:** **Analysis of each conserved sequence of four genes of ISAV**.

**Genomic segment**	**Size of genomic segment (bp)**	**Nucleotide position of conserved sequence (start-term)**	Sequence identity %^*^
Nucleoprotein	1800	1076–1575	89.2
Fusion	1367	335–834	99.5
Hemagglutinin	1182	1–500	99.4
Matrix	861	95–594	92.7

#### Detection and quantitation of dsRNA produced in *E. coli* HT115 (DE3)

The production of each dsRNA was observed in a 1% w/v agarose gel as one band of approximately 500 bp. These bands were purified, and conventional PCR reactions of the internal regions of the conserved sequences were performed. The expected size was observed for each purified dsRNA, with bands of 205, 224, 182, and 200 bp for NP, F, HE, and M, respectively, demonstrating successful expression (Supplementary Figure 1A). Furthermore, dsRNAs were visualized and quantified in a bioanalyzer (Supplementary Figure 1B). The concentration of each band was used to calculate the number of dsRNA copies produced by each milligram of bacteria. Values of 2.44 × 10^13^, 6 × 10^13^, 2.41 × 10^13^, and 7.87 × 10^13^ copies of dsRNA/mg bacteria were obtained for NP, F, HE, and M, respectively.

### Delivery of dsRNA into ASK cells

#### Co-localization assay of *E. coli* HT115 (DE3) overexpressing GFP in ASK cells

**Figure 1:**
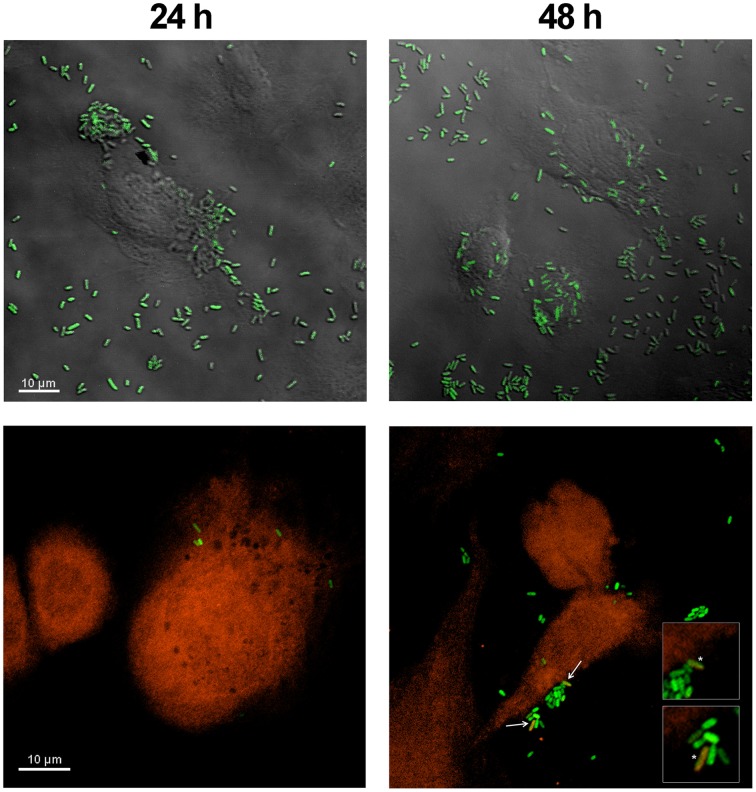
**Co-localization assay of *E. coli* HT115 expressing GFP in ASK cells**. Green *E. coli* were added to ASK cells stained with fluorescent orange dye. Phase contrast (upper panel) and fluorescence (lower panel) confocal microscopy images were taken at 24 h (left) and 48 h (right). Bacteria were visualized as orange/yellow when they co-localized with the orange fluorescent probe in the cell cytoplasm, as indicated with arrows at 48 h in the right bottom panel and an asterisk in the enlarged panel. Scale bars represent 10 μm.

*E. coli* HT115 (DE3) overexpressing GFP was used in an attempt to evaluate the endocytosis of these bacteria after treatment with formaldehyde. Green *E. coli* HT115 (DE3) were observed near ASK cell membranes at 24 h post-incubation with bacteria (Figure 1, upper left panel) but were not uptaken, as observed when a cytoplasmic probe was used in co-localization assays (Figure 1, bottom left panel). At 48 h post-incubation, a few bacteria (1 or 2/cell) were present as an orange/yellow mark in conjunction with GFP fluorescence, indicating co-localization with ASK cytoplasm (Figure 1, arrows and asterisks in bottom right panel), suggesting the internalization uptake of bacteria. These data were confirmed using a Z-plane reconstruction (data not shown). This phenomenon was also observed at 96 h post-incubation with bacteria, although no increase in the number of uptaken bacteria per cell was observed (data not shown).

### Effect of dsRNA

#### Effect of dsRNA targeting NP, F, HE, or M on the expression of their gene targets

**Figure 2:**
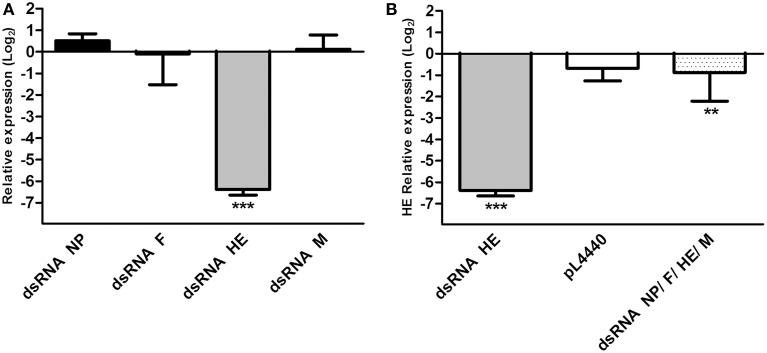
**Relative expression of viral mRNA in infected ASK cells treated with different dsRNAs**. **(A)** Relative viral mRNA expression of NP, F, HE, and M in ASK cells infected with ISAV HPR35 and treated with HT115 *E. coli* carrying dsRNA against NP (dsRNA NP), F (dsRNA F), HE (dsRNA HE), or M (dsRNA M). Data were compared with viral mRNA expression of infected but non-treated ASK cells and expressed as fold change in log_2_. **(B)** Relative expression of HE mRNA in ASK cells infected with ISAV HPR35 and treated with HT115 *E. coli* carrying dsRNA against HE (dsRNA HE), a mixture of the four *E. coli* strains carrying each of the dsRNAs (dsRNA NP/F/HE/M) or *E. coli* carrying the empty vector (pL4440) compared with viral mRNA expression in infected but non-treated ASK cells. Data are expressed as fold change in log_2_. Bars represent the average results (*n* = 3), and the error bars represent standard errors of the means (SEM). (^***^) indicates significant differences between treated, infected ASK cells vs. untreated, infected ASK cells, *p* < 0.05. ^**^indicates significant differences between NP/F/HE/M-mix treated, infected ASK cells vs. HE treated, infected ASK cells, *p* < 0.05.

To evaluate whether dsRNAs produced by *E. coli* HT115 were able to decrease the expression of viral genes, infected ASK cells were treated with bacteria expressing each dsRNA (NP, F, HE, or M) independently, expressing all of the dsRNAs together in equal concentrations (NP/F/HE/M) or not expressing dsRNA. For purposes of comparison, the relative expression of viral genes in control (ISAV HPR35) and treated ASK cells (ISAV HPR35 + dsRNA) were measured. Relative amounts of each viral transcript are expressed as the fold change in NP, F, HE, or M in infected cells treated with bacteria carrying the antiviral, compared to infected cells without antiviral treatment. All data were normalized to β-actin expression. The expression levels of NP, F, and M mRNAs were not significantly affected by treatment with their specific dsRNAs (Figure 2A). However, mRNA expression of the gene encoding HE was significantly reduced (six-fold change in log_2_) by dsRNA against HE (Figures 2A,B). HE mRNA expression was not affected when infected ASK cells were treated with empty vector (no dsRNA, pL4440) and decreased to a lesser degree (1-fold change in log_2_) when the cells were treated with a combination of equal parts of the bacteria carrying their corresponding dsRNAs (dsRNA NP/F/HE/M) (Figure 2B).

#### Effect of dsRNA against HE on HE protein expression

**Figure 3:**
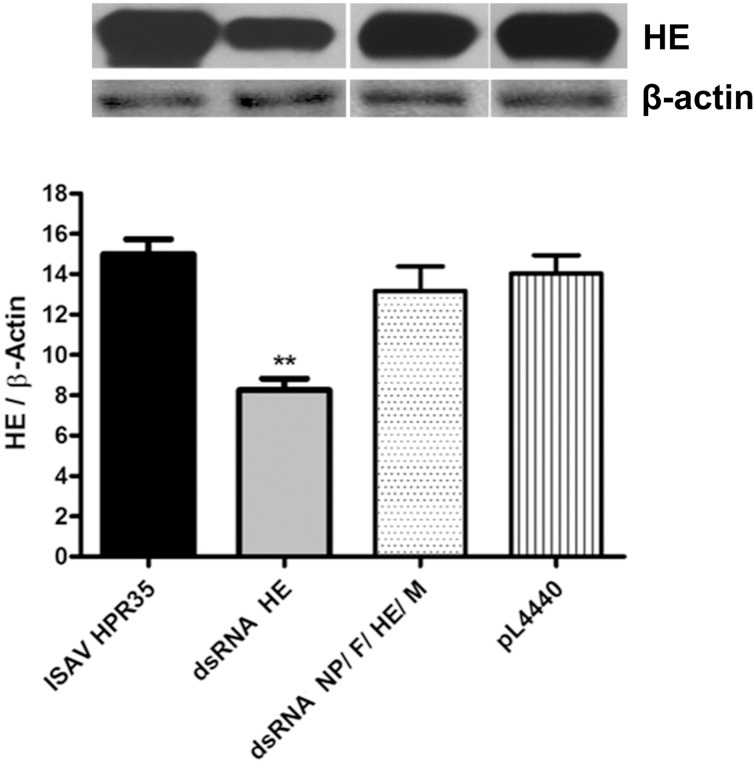
**Expression of HE protein in infected ASK-cells treated with dsRNA**. Lysates from ASK cells infected with HPR35 ISAV, infected ASK cells treated with *E. coli* HT115 carrying dsRNA against HE (dsRNA HE), the dsRNA NP/F/HE/M mixture or empty vector (pL4440) were analyzed by western blot using HE-antibody for viral protein detection. All data were normalized to β-actin expression (HE/β-Actin). Bars represent the average result (*n* = 3), and the error bars represent standard errors of the means (SEM). (^**^) indicates a significant difference between infected ASK cells treated with dsRNA against HE (dsRNA HE) vs. ASK infected cells (ISAV HPR35), and infected ASK cells treated with the empty vector (pL4440), *p* < 0.05.

HE protein expression was significantly decreased by 45% in infected ASK cells using dsRNA against HE (dsRNA HE) compared with infected and non-treated cells (ISAV HPR35) (Figure 3), by western blot analysis. On the other hand, HE protein expression in infected ASK cells treated with bacteria carrying equal amounts of dsRNA against NP, F, HE, and M (dsRNA NP/F/HE/M) or with empty bacteria (pL4440) was unaltered compared with the ISAV-infected control (ISAV HPR35).

#### The effect of HE dsRNA on the ISAV viral load in ASK cultures

**Figure 4:**
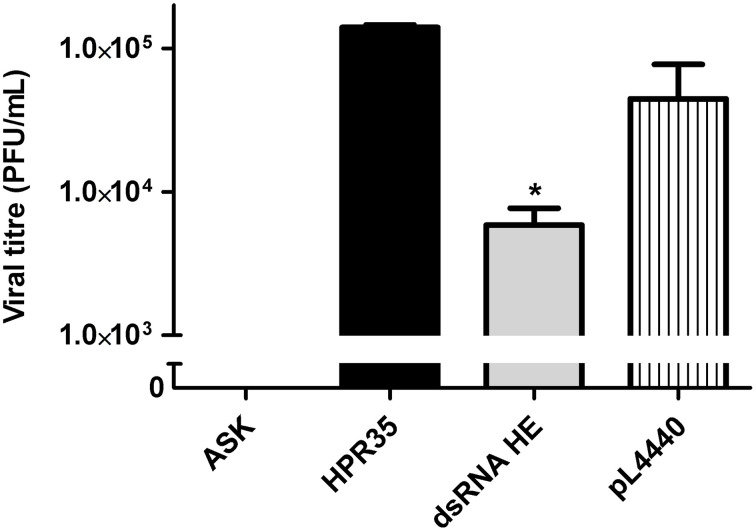
**Effect of HE dsRNA on viral titer (PFU/mL) in the supernatants of infected ASK cells**. Viral titers (PFU/mL) of ISAV in the supernatants of ASK cells infected with ISAV HPR35 (HPR35), infected ASK cells treated with *E. coli* HT115 carrying dsRNA against HE (dsRNA HE) or empty vector (pL4440). The supernatant of uninfected cells was added as an assay control (ASK). Bars represent the average result (*n* = 3), and the error bars represent standard errors of the means (SEM). (^*^) indicates a significant difference between HPR35-infected ASK cells treated with dsRNA against HE (dsRNA HE) vs. infected ASK cells (HPR35), *p* < 0.05.

To assess whether the decrease in the HE protein level correlated with a lower number of infectious viral particles, a viral plaque assay was performed. The viral titer (PFU/mL) in the supernatant of infected ASK cells treated with dsRNA against HE (dsRNA HE; 5.9 × 10^3^ PFU/mL) was 24-fold lower than the viral titer in supernatants of non-treated cells (HPR35, 1.4 × 10^5^ PFU/mL). Bacteria carrying no dsRNA (pL4440) had no significant effect on ISAV viral titer (Figure 4). An uninfected cell control (ASK) was added to verify that cells did not form lysis plaques for other reasons (Figure 4).

#### The effect of HE dsRNA on the CPE of ISAV in ASK cultures

**Figure 5:**
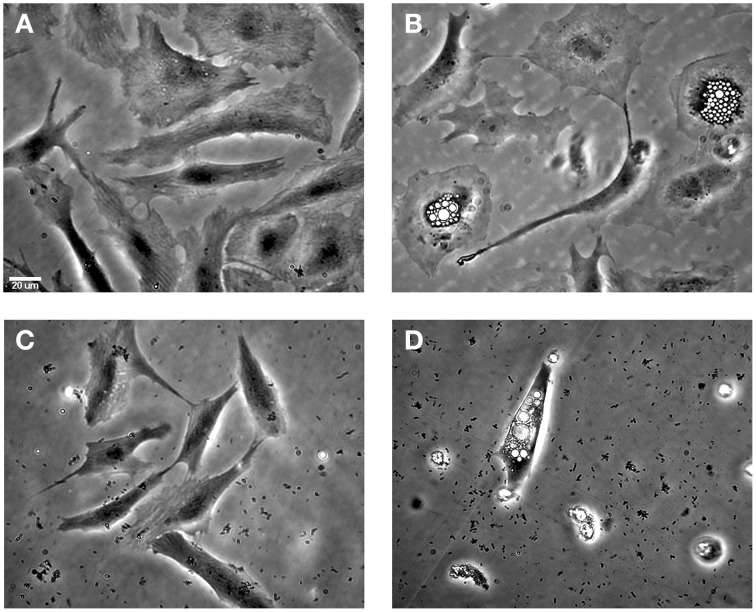
**The effect of HE dsRNA on induced CPEs in infected ASK cells**. **(A)** Uninfected ASK cells at 11 days post-infection **(B)** ASK cells infected with HPR35 ISAV without treatment and **(C)** with the addition of *E. coli* carrying dsRNA against HE. **(D)** Infected ASK cells and treatment with *E. coli* HT1115 carrying empty vector pL4440 was included as a control. The CPEs observed in ASK cells infected as described were vacuolated cells and monolayer detachment. The scale bar represents 20 μm.

Microscopic observations revealed characteristic phenotypic changes when ASK cells were infected with ISAV HPR35. At day 11 post-infection, infected cells showed an extensive CPE with numerous vacuolated and stellated-shaped cells with partial monolayer detachment (Figure 5B). In contrast, when infected cells were treated with HE dsRNA, the cells showed decreased CPE, no vacuolated cells and less monolayer detachment (Figure 5C). In infected cells treated with bacteria expressing no dsRNA against HE, the cellular damage observed was similar to that upon infection with ISAV with no additional treatment (Figure 5D). As a control, ASK cells after 11 days of cultivation are shown in Figure 5A.

#### The effect of dsRNAS on Mx expression in ASK cultures

To evaluate the ability of dsRNAs produced by *E. coli* HT115 to induce expression of Mx, a marker of the interferon pathway, uninfected ASK cells were treated with bacteria expressing each dsRNA (NP, F, HE, or M) independently or expressing no dsRNA (empty vector pL4440). All gene expression data were normalized to β-actin expression. The expression levels of Mx mRNAs were significantly induced at similar extent by all dsRNAs (Supplementary Figure 2).

## Discussion

RNAi is a promising methodology for developing new therapeutic and antiviral strategies (Gavrilov and Saltzman, 2012). RNAi has been suggested for the development of therapies aimed at the treatment of viral diseases and parasites in aquatic organisms (Lima et al., 2013), with successful reports on studies using RNAi against numerous viruses that cause problems in aquaculture. Sarathi et al. (2008b) were the first to show the antiviral ability of bacterially synthetized dsRNAs. They revealed that Vp28 dsRNA produced in bacteria was able to silence VP28 gene expression in WSSV. Similarly, others studies *in vitro* (Kim and Kim, 2011; Kim et al., 2012) have revealed that it is possible to control the virus causing viral hemorrhagic septicemia (VHSV), a single-stranded RNA genome virus that causes important viral diseases in a wide variety of wild and cultured fish species worldwide (Kim et al., 2012), using dsRNA antivirals in EPC (Epithelioma papulosum cyprinid) and CHSE214 (chinook salmon embryo) cells. Additionally, Kim and colleagues showed that long dsRNA antivirals are able to reduce the expression of the VHSV G gene, which encodes a glycoprotein, in a manner that is dependent on RNAi because they do not activate the interferon pathway (Kim et al., 2012). Later studies showed the feasibility of using RNAi *ex vivo* to inhibit the viral replication of cyprinid herpesvirus-3 (CyHV-3) in cultured cells (Gotesman et al., 2014). Some of these successful studies support the methodology utilized in this study, which was based on the use of long bacterially synthesized dsRNAs, to inhibit replication of ISAV *ex vivo* by impairing the expression of viral proteins. To our knowledge, this is the first experimental approach using RNAi against the ISA virus. We searched conserved genomic regions within sequences of Chilean isolates that were available in the database (Table 1). Despite the few available sequences found for the NP and M genes, one conserved genomic region of 500 bp was detected in all genes of interest. Once we had designs for dsRNA, we used *E. coli* HT115 (DE3) for production and delivery of dsRNA because it is a good option for large-scale production and *in vivo* delivery in aquaculture systems (Sarathi et al., 2008a). These bacteria have been widely used for production and delivery of dsRNAs in other study models, such as *C. elegans* (Timmons and Fire, 1998; Kamath et al., 2001; Timmons et al., 2001). However, we observed that only one or two bacteria co-localized with the cytoplasm of ASK cells in our co-localization assay, suggesting that few bacteria were uptaken. We confirmed these observations performing PCR analysis of viral HE sequence using DNA preparations from ASK cells treated with attenuated bacteria (Supplementary Figure 3). These results agree with an observation by Simon and Leong (2002), who rarely observed gene transfer into CHSE-214 cells, another salmon-derived cell line, when using *E. coli* as a vector. Additionally, Grillot-Courvalin and colleagues observed that fewer than 0.1% of CHO cells (derived from Chinese hamster ovaries) received gene transfer when *E. coli* was used as a vector. However, when invasive *E. coli* were used, 6% of cells were found to be positive, indicating successful gene transfer (Grillot-Courvalin et al., 1998). Invasive *E. coli* have been found to act as a good gene delivery system in both phagocytic and non-phagocytic mammalian cells (Grillot-Courvalin et al., 1999), showing that using invasive non-pathogenic bacteria is a plausible strategy by which to obtain better uptake and delivery results. Despite the low number of uptaken bacteria in our experiments, the silencing efficiency of the four bacterially expressed dsRNAs against the NP, F, HE, and M genes was investigated. The dsRNA against HE was found to significantly reduce mRNA expression. This result indicates that despite the low number of bacteria entering cells, the dsRNA input was sufficient to act in the ASK cells and specifically inhibit the expression of HE mRNA. It draws attention the fact of the mRNA expression of the gene encoding HE was significantly reduced (six-fold change in log_2_) by dsRNA against HE, but it decreased to a lesser degree (1-fold change in log_2_) when the cells were treated with a combination of equal parts of the bacteria carrying their corresponding dsRNAs (dsRNA NP/F/HE/M). Similarly, HE protein expression was significantly decreased by 45% in infected ASK cells using dsRNA against HE (dsRNA HE) compared with infected and non-treated cells (ISAV HPR35) but HE protein expression in infected ASK cells treated with bacteria carrying equal amounts of dsRNA against NP, F, HE, and M (dsRNA NP/F/HE/M) was unaltered compared with the ISAV-infected control. These findings strongly suggest a dose-dependency effect of dsRNA against HE in the assays. In addition, the absence of an effect on the HE mRNA level during the treatment with bacteria carrying empty L440 suggest that the inhibition of HE gene transcription was a response of the RNAi machinery induced by dsRNA against HE. However, this response seems to be combined with non-specific effects, since the expression of Mx gene (involved in the interferon pathway) is activated when non-infected ASK cells are treated with attenuated bacteria expressing dsRNA (data not shown). The fact that some dsRNA induces sequence specific antiviral activity in addition to non-specific immunity is not new, and it has been previously observed by other authors (LaFauce and Owens, 2009). dsRNA against HE was able to induce specific effect decreasing HE mRNA and consequently, decreasing HE protein expression. In contrast, dsRNA against NP, F, M had no effect decreasing the expression of their target viral proteins. This could be due to a limited uptake of bacteria by ASK cells. However, bacteria carrying the other dsRNA (against NP, F, and M) are also isogenic; among them the only difference is the insert, even insert size is ~500 bp in all cases. Furthermore, the number of dsRNA copies per mg of bacteria is very similar ~10^13^ copies of dsRNA/mg bacteria in all cases. For this reason, we believe unlikely that differences in the antiviral effects were based on different bacterial uptake. The other reason may be explained by differences in the insert. It was reasonable that the target mRNAs for the other dsRNA molecules tested (NP, F, and M) were not silenced because other studies have shown that the degree of protection afforded by specific dsRNAs, varies between different targeted viral genes. The degree of protection conferred by dsRNA is well-known to depend on the target viral gene (Robalino et al., 2005). In this work, we selected ISAV structural proteins as candidates for silencing. Some works have compared differences in silencing non-structural and structural protein genes and found no clear differences (Flores-Jasso et al., 2004; Sanjuktha et al., 2012). However, Sanjuktha and colleagues showed that the function of the encoded protein is a more important criterion for selection (Sanjuktha et al., 2012). Moreover, the gene silencing effect of dsRNA is likely to vary substantially with the targeted position on the mRNA (Luo and Chang, 2004). Robalino et al. (2005) reported that different mRNAs were differentially susceptible to sequence-dependent targeting. Until now, the reasons for the differences in RNAi efficacy have not been well-understood, but they may involve primary sequence and secondary structure properties of dsRNA and targeted viral mRNAs. Additionally, the binding of viral and/or cellular proteins to the targeted RNA sequence may prevent recognition by the RNA-induced silencing complex (Hasnoot et al., 2003).

The strong decrease in the HE mRNA level was reflected in a 45% decrease in protein expression, although the significant reduction in mRNA when all antivirals were used together, was not reflected in a significant protein reduction. These results likely occurred due to the high stabilization of the remaining mRNA, as has been shown in other viruses, such as equine infectious anemia virus (Martarano et al., 1994) and rabies virus (Palusa et al., 2012). Nonetheless, the decrease in the protein level in 45%, was associated with a strong decrease in the viral titer, which decreased by 24-fold. This effect was probably due to the combined response of RNAi pathway with non-specific effects induced in a sequence-independent manner, since the expression of Mx gene is activated in uninfected ASK cells treated with dsRNA against HE (Supplementary Figure 2). However, Mx expression was induced at similar extent by dsRNA HE and the other dsRNA molecules, which had no antiviral effect, hence, we suggest that the inhibition of HE gene transcription and the decrease in the viral titer occur mainly due to specific effects of dsRNA HE treatment. Concomitantly, the lower number of infective viral particles was reflected in a minor CPE in infected ASK cells, and cells with hallmark morphologic changes (vacuolated cells) and monolayer detachment associated with viral infection were not observed. These effects are not specific to ISAV because the same phenomenon has been observed when gene expression is silenced *ex vivo* using RNAi against other viruses. In the case of goatpox, a decline in the number of infective viral particles was reflected in a reduced CPE in Vero cells (Zhao et al., 2012). A similar result was observed when RNAi was used against the equine encephalitis virus in Vero cells and U87MG; all effective RNAi silencing of the target gene decreased the CPE associated with viral infection (Lundberg et al., 2013).

The results obtained in this work using an *ex vivo* model support for the first time the possible use of RNAi against the ISA virus. However, the administration of RNAi *in vivo* is still a substantial challenge. Some reports have already studied the efficacy of RNAi administered *in vivo* (LaFauce and Owens, 2009). These studies have shown that RNAi confers protective effects by slightly reducing the viral titer of *Penaeus merguiensis* densovirus, and it has a large effect on the prevention of mortality in treated crickets (LaFauce and Owens, 2009). This fact indicates that protective non-specific effects likely occur in some instances of RNAi, as we also observed with dsRNA against HE.

## Conclusion

In light of the results presented, we demonstrated that long dsRNA against HE elicits the RNAi process in salmon cells, and these dsRNA molecules are able to reduce ISAV replication and viral load in infected ASK cells. Although it seems that dsRNA also induces non-specific effects of the immune response. This strategy could still be improved and optimized, leading us to design new methodologies in the future using *in vivo* models to provide an efficient tool for ISAV control in cultured fish.

## Author contributions

KG drafted the manuscript. KG, SRA, and AD made cell cultures, real time PCR, WB, and ECP assays and they analyze the results. SRA and SRC made co-localization assays of bacteria in culture cell. SRC and GH made viral plaque assays. SRA also made statistical analysis. RE participated analysis of results. JR conceived the study and participated in its design and coordination. All authors read and approved the final manuscript.

### Conflict of interest statement

Jaime Romero, Katherine García, and Álvaro Díaz hold a patent, number 1473-2014. Requested patent 2014-01473 PCT Chile. The authors declare that the research was conducted in the absence of any commercial or financial relationships that could be construed as a potential conflict of interest.
