# Supplementary material for: Inactivated E. coli transformed with plasmids that produce dsRNA against infectious salmon anemia virus hemagglutinin show antiviral activity when added to infected ASK cells
Source: Front Microbiol. 2015 Apr 16;6:300. doi: 10.3389/fmicb.2015.00300 (PMC4399331; doi:10.3389/fmicb.2015.00300)
Supplement: Supplementary file 1 [file Table1.DOCX]

**Supplementary Table 1.** Conserved regions identified in four ISAV genes used to dsRNA design.

| **Genomic segment** | **Conserved consensus sequence** |
| --- | --- |
| **3 (NP)** | CTCTGTGCACAAGCTTTCTGATTGACCCACCTAGAACAACCAAATGCTTCATTCCACCTATCTCTAGTCTCTTGATGTACATTCAGGACGGAAATTCAGTGTTGGCAATGGACTTCATGAAGAACGGAGAAGATGCTTGCAGGATCTGCAGAGAGGCAAAGCTGAAGGTTGGAGTGAACGGCACATTCACAATGTCTGTGGCTAGAACATGTGTAGCTGTGTCAATGGTTGCAACAGCATTCTGTTCAGCAGATATCATCGAGAATGCAGTTCCCGGCTCAGAAAGGTACAGGTCAAACATCAAGGCAAACACAACCAAACCCAAGAAGGACTCAACATACACAATCCAAGGGCTGAGGCTGTCCAATGTGAAGTACGAGGCAAGACCTGAAACATCTCAAAGCAACACAGATCGAAGCTGGCAAGTGAATGTCACAGACAGTTTCGGAGGGCTAGCGGTTTTCAACCAAGGCGCTATCAGGGAGATGCTTGGAGATGGA |
| **5 (F)** | CCAAATGCGGGAGGAAAGGATTAAAAGTTTTCATCTGCGGAGGTACAACAGGTTATGTAACCAGAGGATGTCCACCGGAGGAATGCAGGGGGAGGAAAGGAAGGATGATGTCTCTAGAACCAACTGCAGACTGTGGAGTGGAAAAAGGCTTTACAACGGAAAGGATTAAGACTGGGAAGGTGGACTTGGATAGCTGTTGCACTCAGCATGGATGTACAAAAGGGATTAGGGTGGAGGTTCCATCGCCTGTACTGGTATCGGCCAAATGCAATGAAATTTCATTCAGAGTAGTGCCGTTCCATTCTGTACCAGACAGGCTAGGGTTCGCTAGAACTAGTTCTTTTACACTAAGAGCCGGCCTCGCTAACCARCATGGATGGTCTAAATACAACTTCAACAAAGGGAAATCAGCTAATGACATTATCTCCGACCAGAGAGCATTCCCAGGAGAAGAGTTCATCAAATGCTGTGGATTTACTTTGGGGATCGGAGGTGCTTGG |
| **6 (HE)** | GGGGGGTGCTGCAGGCCAAAAACGGAAATGGACTTTTGAAGCAGATGAGTGGAAGGTTTCCAAGTGACTGGTACACACCTACTACAAAGTACCGGATCCTATACTTGGGAACCAATGACTGCACTGACGGACCTACTGACATGATCATCCCAACTTCGATGACACTGGACAACGCGGCAAGGGAGCTGTACCTGGGAGCATGCAGGGGAGACGTGAGAGTGACGCCTACATTTGTGGGAGCAGCAATTGTTGGACTTGTTGGACGAACAGACGCAATTACCGGTTTTTCGGTGAAGGTGTTGACTTTCAACAGCYCTACAATTGTAGTGGTGGGATTGAATGGAATGTCCGGAATCTACAAGGTCTGCATTGCAGCAACATCTGGGAATGTGGGAGGAGTGACACTTATCAACGGATGCGGATATTTCAACACACCTTTGAGGTTTGACAATTTCCAAGGACAAATCTACGTGTCAGACACCTTTGAAGTGAGGGGAACC |
| **8 (M)** | AAGAGGCCAGAAGTAGTCTACGCAATGGGAGTGCTACTTACACTTGGCGGGGAAAGCGGATTGTGTGTAGAGTTCCAGGCTCCAGAAGGGAAAATGGTGAAAGTCAAAACCTTGAACCAATTGGTGAACGGAATGATCAGTCGAGCGACGATGACTCTCTACTGTGTGATGAAAGATCCACCGTCTGGAAGCATGGCGACACTGATGAGGGATCACATCAGAAACTGGCTGAAGGAAGAGTCAGGATGCCAAGACGCGGATGGTGGAGAGGAAAAGTGGGCAATGGTGTATGGTATGATTTCACCAGACATGGCGGAGGAGAAGACGATGTTGAAGGACCTGAAGACAATGCTACACAGCAGGATGCAGATGTATGCTCTAGGAGCGAGTTCGAAAGCCCTGGAAACTTTAGAAAAGGCCATCGTCGCTGCAGTTCATCGACTTCCGGCATCCTGCTCGACAGAGAAGATGGTGCTCCTGGGGTACCTGAGATAAGCCCT |
