# Supplementary material for: Inactivated E. coli transformed with plasmids that produce dsRNA against infectious salmon anemia virus hemagglutinin show antiviral activity when added to infected ASK cells
Source: Front Microbiol. 2015 Apr 16;6:300. doi: 10.3389/fmicb.2015.00300 (PMC4399331; doi:10.3389/fmicb.2015.00300)
Supplement: Supplementary file 2 [file Presentation1.PDF]

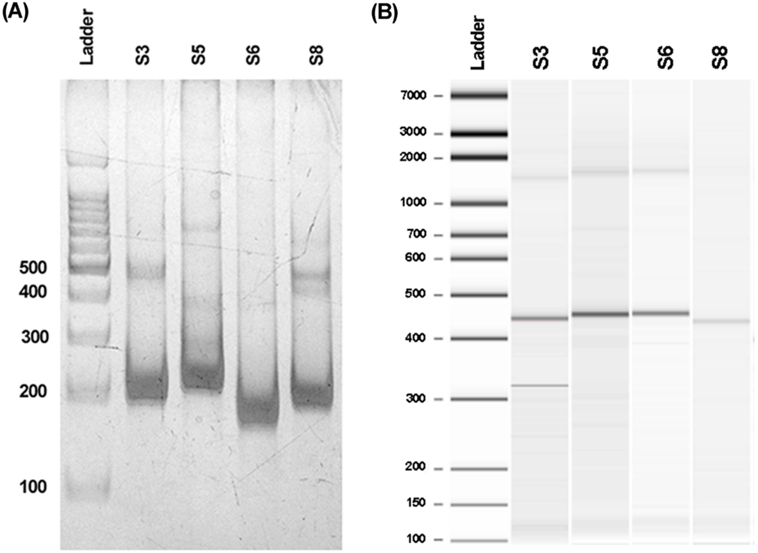

Figure 1 supplementary: dsRNA detection and quantification. (A) Conventional PCR products of the internal regions of dsRNAs, visualized in 8% polyacrilamide gel. Expected size was observed for each purified dsRNA, with bands of 205, 224, 182 and 200 bp for S3 (NP), S5 (F), S6 (HE) and S8 (MP), respectively. **(B)** Band of dsRNA were visualized between 400 and 500 bp, and quantified using the DNA 1000 LabChip kit on a Bioanalyzer Agilent 2100.
