# Supplementary material for: Inactivated E. coli transformed with plasmids that produce dsRNA against infectious salmon anemia virus hemagglutinin show antiviral activity when added to infected ASK cells
Source: Front Microbiol. 2015 Apr 16;6:300. doi: 10.3389/fmicb.2015.00300 (PMC4399331; doi:10.3389/fmicb.2015.00300)
Supplement: Supplementary file 3 [file Presentation2.PDF]

Supplementary Figure 2.

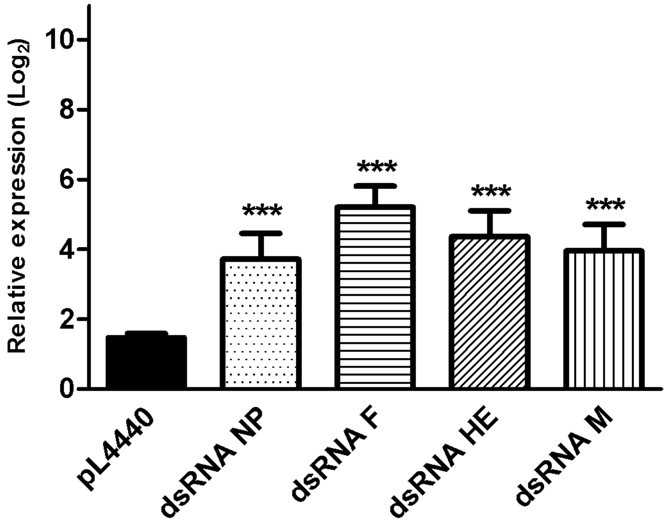

Supplementary Figure 2. **Expression of Mx in ASK-cells treated with different dsRNA.** Relative mRNA expression of Mx in uninfected ASK cells treated with HT115 *E. coli* carrying empty vector (pL4440) or dsRNA against NP, F, HE or M respectively. Data were normalized with β-actin mRNA expression and analyzed with Rest 2009 software to determine if antiviral dsRNA production induces Mx expression in comparison to empty vector. *** = p>0.01.
