# Supplementary material for: Inactivated E. coli transformed with plasmids that produce dsRNA against infectious salmon anemia virus hemagglutinin show antiviral activity when added to infected ASK cells
Source: Front Microbiol. 2015 Apr 16;6:300. doi: 10.3389/fmicb.2015.00300 (PMC4399331; doi:10.3389/fmicb.2015.00300)
Supplement: Supplementary file 4 [file Presentation3.PDF]

**Supplementary Figure 3.-**

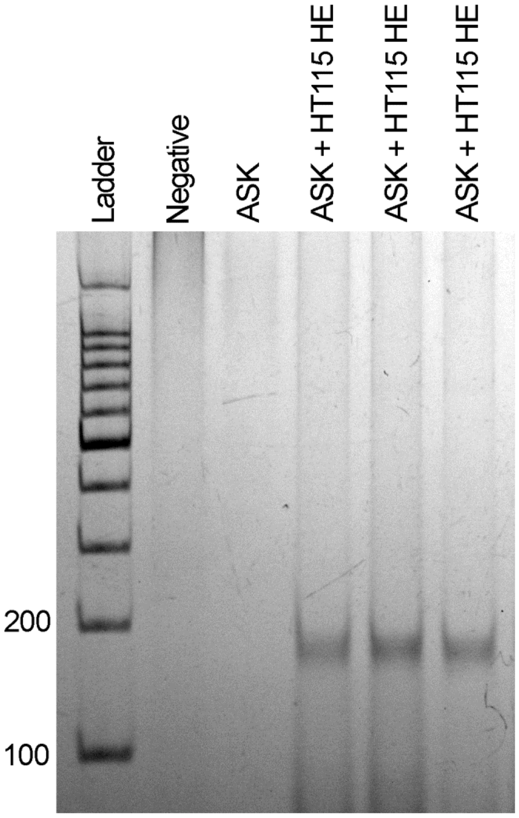


**Supplementary Figure 3.- Electrophoresis of HE to detect the internalization of HT115 HE in ASK cells**. The DNA extracted from ASK cells co-cultured with HT115 HE at 48 h was utilized to PCR assay using HE primers. The amplicon of HE only is observed in ASK cells co-culture with bacteria. Similar results were observed at 24 h.
